# Polyploidy in myelofibrosis: analysis by cytogenetic and SNP array indicates association with advancing disease

**DOI:** 10.1186/1755-8166-6-59

**Published:** 2013-12-17

**Authors:** Nisha R Singh, Christine M Morris, Mary Koleth, Kelly Wong, Christopher M Ward, William S Stevenson

**Affiliations:** 1 Northern Blood Research Centre, Kolling Institute, University of Sydney, Sydney, Australia; 2 Departments of Cytogenetics and Haematology, Pathology North, Royal North Shore Hospital, Sydney, NSW, Australia; 3 Department of Pathology, Cancer Genetics and Cytogenetics, University of Otago Christchurch, Christchurch, New Zealand

**Keywords:** Gain of 1q, Myelofibrosis, Tetraploidy, Polyploidy, SNP array

## Abstract

**Background:**

Myelofibrosis occurs as primary myelofibrosis or as a late occurrence in the evolution of essential thrombocythaemia and polycythaemia vera. It is the rarest of the three classic myeloproliferative neoplasms (MPN). Polyploidy has only rarely been reported in MPN despite the prominent involvement of abnormal megakaryocytes. The use of peripheral blood samples containing increased numbers of haematopoietic progenitors has improved the output from cytogenetic studies in myelofibrosis and together with the use of single nucleotide polymorphism arrays (SNPa) has contributed to an improved knowledge regarding the diverse genetic landscape of this rare disease.

**Results:**

Cytogenetic studies performed on a consecutive cohort of 42 patients with primary or post ET/PV myelofibrosis showed an abnormal karyotype in 24 cases and of these, nine showed a polyploid clone. Six of the nine cases showed a tetraploid (4n) subclone, whereas three showed mixed polyploid subclones with both tetraploid and octoploid (4n/8n) cell lines. The abnormal clone evolved from a near diploid karyotype at the initial investigation to a tetraploid karyotype in follow-up cytogenetic analysis in four cases. In total, six of the nine polyploid cases showed gain of 1q material. The remaining three cases showed polyploid metaphases, but with no detectable structural karyotypic rearrangements. Three of the nine cases showed chromosome abnormalities of 6p, either at diagnosis or later acquired. SNPa analysis on eight polyploid cases showed additional changes not previously recognised by karyotype analysis alone, including recurring changes involving 9p, 14q, 17q and 22q. Except for gain of 1q, SNPa findings from the polyploid group compared to eight non-polyploid cases with myelofibrosis found no significant differences in the type of abnormality detected.

**Conclusions:**

The study showed the use of peripheral blood samples to be suitable for standard karyotyping evaluation and DNA based studies. The overall profile of abnormalities found were comparable with that of post-MPN acute myeloid leukaemia or secondary myelodysplastic syndrome and cases in the polyploidy group were associated with features of high risk disease. The above represents the first documented series of polyploid karyotypes in myelofibrosis and shows a high representation of gain of 1q.

## Background

The classic myeloproliferative neoplasms (MPN) encompass three disease subsets, including polycythaemia vera (PV), essential thrombocythaemia (ET) and primary myelofibrosis (PMF). PMF occurs at an incidence of 0.3-1.5 per 100,000 and is characterised clinically by anaemia, splenomegaly and progressive bone marrow (BM) fibrosis [1]. Thrombocytopenia or thrombocytosis is frequent [2]. Morphologically, PMF patients typically show a leukoerythroblastic blood film, elevated numbers of circulating CD34+ cells and the presence of prominent, abnormal, dysplastic megakaryocytes in the BM [3]. Approximately 5% of ET and 20% of PV patients progress to a secondary myelofibrosis (sMF), usually after a 15–20 year follow up period. PMF may also transform to acute leukaemia in 8-23% of cases in the first 10 years post-diagnosis [4].

Abnormal karyotypes occur in approximately 50% of PMF cases. Chromosome aberrations including +1q, +8, del(12p), del(13q) and del(20q) are commonly reported across all three MPN [5]. These structural chromosome abnormalities and a variety of molecular defects including gene mutations affecting *JAK2, MPL, TET2, LNK, EZH2, NF1, IDH1, IDH2, CBL, ASXL1, IKAROS* and *NF-E2* are prevalent but not specific to any subset of MPN and have been described in other myeloid disorders [6,7]. The underlying molecular pathogenesis driving fibrosis remains unknown despite multiple studies to date attempting to identify a common genetic defect [8]. This has contributed to difficulties in developing effective targeted therapies.

Polyploidy refers to an increased number of the complete set of chromosomes and occurs in multiples of the haploid set. Polyploidy may be found as a natural phenomenon in some mammalian cells such as megakaryocytes and hepatocytes or it may occur in relation to a pathological state. Polyploidy in normal megakaryocyte precursors is achieved by endomitosis whereby cells enter mitosis repeatedly during cell cycling, do not complete mitosis but instead re-enter G1 and proceed through S phase and G2/M in repeated cycles. As a result of endomitosis the cell is able to conserve energy while producing the large numbers of anucleate platelets needed for normal haemostasis [9].

Disruption of normal endomitosis influences megakaryocyte ploidy and platelet production and may result in thrombocytopenia or thrombocytosis [10]. Patients with MF show characteristic large, bizarre and dysplastic forms of megakaryocytes that are clonal, but reports of polyploid karyotypes in the literature are rare [11]. Studies suggest a disruption in the normal mechanism for polyploidisation during megakaryocyte growth in PMF leading to an increase in megakaryocyte numbers but with reduced polyploidy [12,13].

The generation of polyploid and in particular tetraploid karyotypes in neoplastic cells may involve different mechanisms such as abortive mitosis, failure of cytokinesis or centrosome amplification. Tetraploidy has been postulated as a precursor in the formation of stable aneuploidy and in the development of chromosome instability in cancer. An additional role in the suppression of tumourigenesis in some scenarios makes tetraploidy a possible therapeutic target [14,15].

In this study, the clinical and molecular cytogenetic characteristics of nine patients showing a polyploid metaphase clone are discussed in detail. In addition, study of the underlying mechanism for formation of the polyploid clones was performed on samples with adequate numbers of viable cells.

## Results

### Patient characteristics

Peripheral blood (PB) and BM samples were obtained from 42 patients with myelofibrosis (MF) associated with MPN between July 2006 and July 2012 (Additional file 1). The median age of the patient cohort was 64 years (range: 44–82 years), with 16 females and 26 males included in the study. Thirty-two individuals were classified as PMF, five had post-polycythaemia vera MF (PPV-MF) and five had post-essential thrombocythaemia MF (PET-MF). At the time of initial sample collection, all patients were in the fibrotic phase of the disease with no evidence of leukaemic transformation.

### Polyploidy in MF associates with a high representation of 1q gain

A polyploid subclone was detected in nine MF patients by karyotype analysis (Case nos. 1–9, Additional files 1 and 2). Seven cases were classified as PMF, one as PPV-MF and one with PET-MF. At the commencement of the study three patients were being treated with hydroxyurea, one with interferon, one with thalidomide and one with aspirin. Three patients were asymptomatic and untreated. Two polyploid groups were noted: a tetraploid (4n) group and a mixed (4n/8n) ploidy group. Polyploidy was detected in both colcemid and vinblastine-colchicine arrested PB cultures.

**Figure 1:**
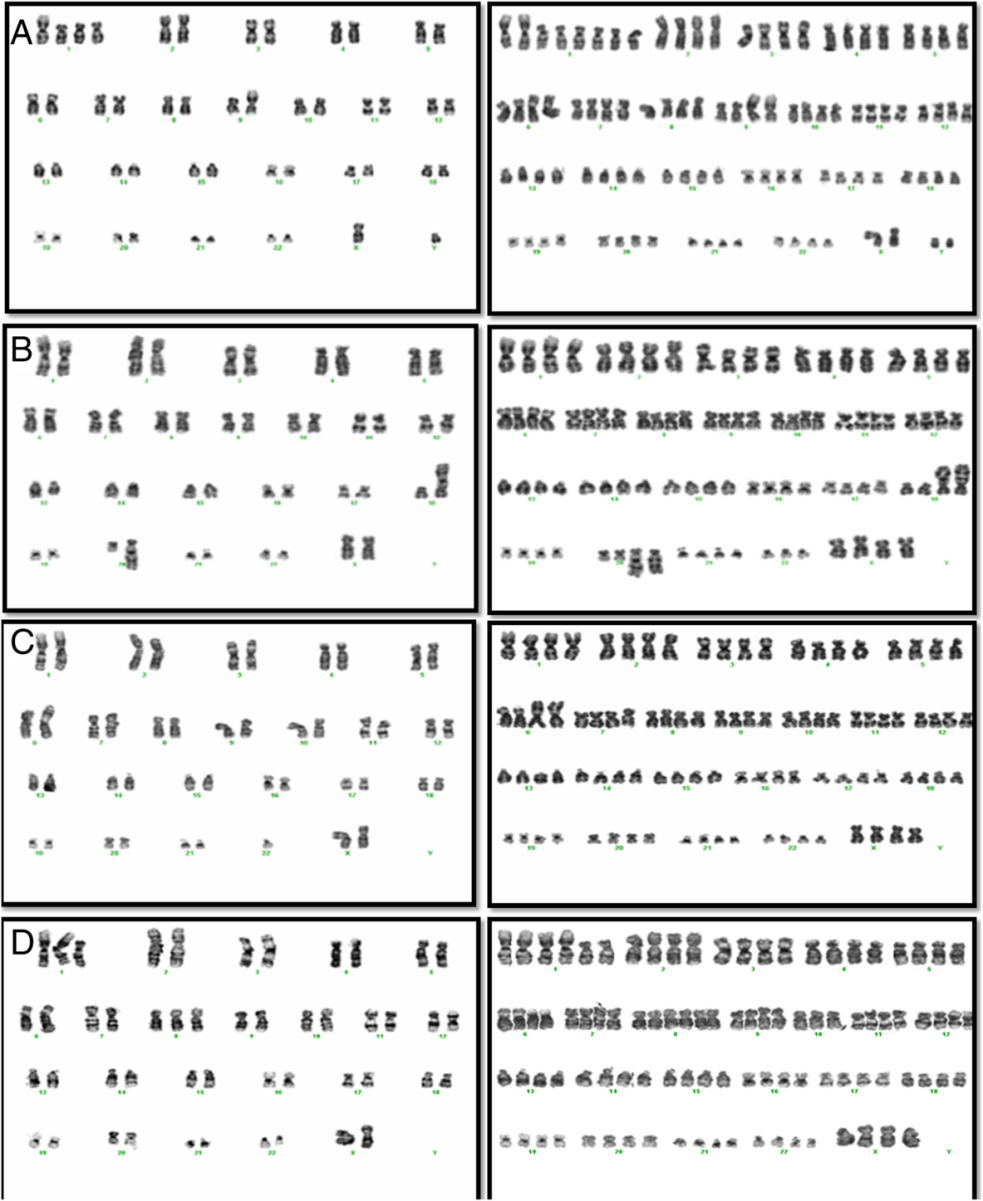
**Karyotypes from Case nos. 1–4 (A - D, respectively) showing unbalanced 1q arm translocations.** Serial tetraploidisation is shown on the right hand karyotype. **A)** Reciprocal translocation t(1;9) with two additional copies of the derivative (1q)t(1;9) with subsequent rearrangement on 6p prior to tetraploidisation. **B)** Two unbalanced translocations showing translocation of 1q with the der(18)t(1;18) and der(20)t(1;20) as well as an interstitial deletion on 13q. **C)** The der(6)t(1;6) resulting in gain of 1q material and loss of distal 6p as the sole karyotypic abnormality (loss of chromosome 20 was random). **D)** Complex karyotype showing the additional derivative (1q) from the der(1;7)(q10;p10) resulting in gain of both 1q and 7p. Gain of 8, pericentric inversion 12 and deletion 22q are also evident. The tetraploid karyotype shows acquisition of the t(6;9)(p22;q34).

Case nos. 1–4 (Additional file 2) showed an abnormal near-diploid clone with gain of 1q derived from an unbalanced translocation. These clones were present at initial diagnosis in two of the patients studied (Case nos. 1, 2), and at first successful cytogenetic investigation in Case no. 4. A sample from the fourth patient (Case no. 3) was not cytogenetically evaluated at diagnosis but showed the der(6)t(1;6)(q21;p21) as the sole karyotypic change on the initial cytogenetic investigation in 2008. Serial cytogenetic analyses indicated that a tetraploid subclone developed subsequently in all four cases after a period of 5–8 months during the term of this cytogenetic study (Additional file 2), and representative karyotypes are shown for each case in Figure 1. One case showed mixed ploidy with an interstitial duplication on 1q (Case no. 5) and one case with a complex karyotype showed the same interstitial dup(1q) in a minor tetraploid cell line (Case no. 6). Case nos. 7–9 showed polyploidy without structural chromosome abnormalities. The size of the polyploid clone ranged from 10% to 65% of metaphases analysed (median: 25%).

Polyploid clones were persistent over time, and confirmed in both PB and BM in five cases (nos. 2, 3, 5, 7 and 8, Additional file 2). Follow up cytogenetic analysis showed further karyotypic abnormalities. One patient showed emergence of a hyperdiploid cell line (Case no. 2), and two patients subsequently acquired minor subclones with possible duplication of 6p (Case no. 1), and the t(6;9)(p22;q34) (Case no. 4) (Figure 1).

### SNP array analysis detects further genomic changes

Single nucleotide polymorphism array (SNPa) analysis was performed on a total of 16 cases. Eight of the nine polyploid cases were studied and results were compared to SNPa on eight MF cases without polyploidy (Additional files 3 and 4). All unbalanced karyotypic abnormalities were detected by SNPa, while minor subclones, balanced rearrangements and polyploidy were not detected using this approach.

### Polyploid cohort

The polyploid samples showed a median of three aberrations per patient (range: 0–8). Twenty six changes in total were detected. Two samples showed no abnormalities by SNPa (Case nos. 7 and 8). For Case nos. 1–3 and 5, no additional copy number changes were detected by SNPa in genomic segments flanking the centromeric region on the 1p arm (Additional file 4). The commonly duplicated region 1q21.1-32.1 contains an estimated 61.81 Mb of DNA (Additional file 4), and 2,807 known genes (http://genome.ucsc.edu, 21/04/2013). Twenty two genes on1q are listed in the Cancer Genome Census including *PDE4DIP, ARNT, NTRK1, PBX1, PRCC, PMX, ABL2, TPR* and *MDM4.*

**Figure 2:**
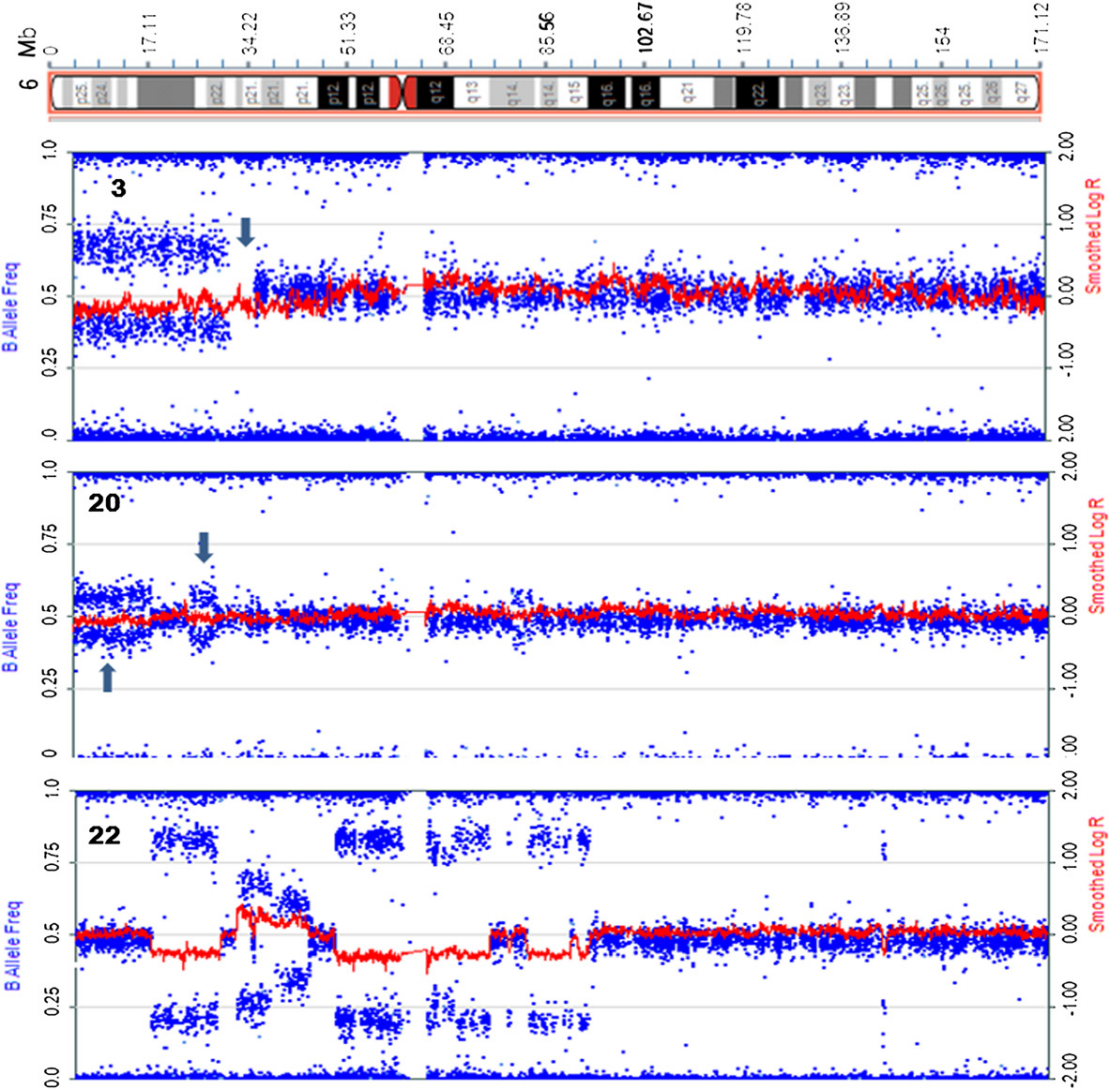
**Abnormalities detected by SNPa on chromosome 6p.** SNPa profiles showing changes in B allele frequency (blue) and LogR ratio (red) on three cases with abnormalities on 6p. Segments showing CN-LOH are denoted by an arrow. From top: Case no. 3, deletion of terminal 6p on the der(6)t(1;6) flanked by a region of CN-LOH; Case no. 20, regions of mosaic CN-LOH occurring at the terminal end as well as interstitially, and Case no. 22, complex changes involving regions of both deletion and amplification from 6p24.1 to 6q14.

Figure 2 shows SNPa profiles of chromosome 6 in three of the 16 SNPa cases. The breakpoint at 6p22.1 associated with the der(6)t(1;6)(q21;p22) in Case no. 3 resulted in a 27 Mb deletion from 6p22.2 to 6pter and encompasses the *DEK* oncogene as well as *JARID2*, a member of the polycomb repressor complex 2 cluster of genes known to be implicated in MPN. A long 4.5 Mb stretch of copy neutral loss of heterozygosity (CN-LOH) was also detected in this case, extending from the 6p22.1 breakpoint toward the centromere to band p21.33 (Additional file 4). This segment houses the chromatin modifier gene *HIST1H4I* and the transcription factor *POUF5I*. A second case (Case no. 1) showed a 3.8 Mb CN-LOH at 6p, extending from 6p21.1 to 6p12.3 (Additional file 4) and encompassing *CENPQ,* involved with chromosome separation at mitosis. The latter patient subsequently acquired a structural karyotypic rearrangement in a subclone involving the more telomeric band 6p25 (Additional file 2).

**Figure 3:**
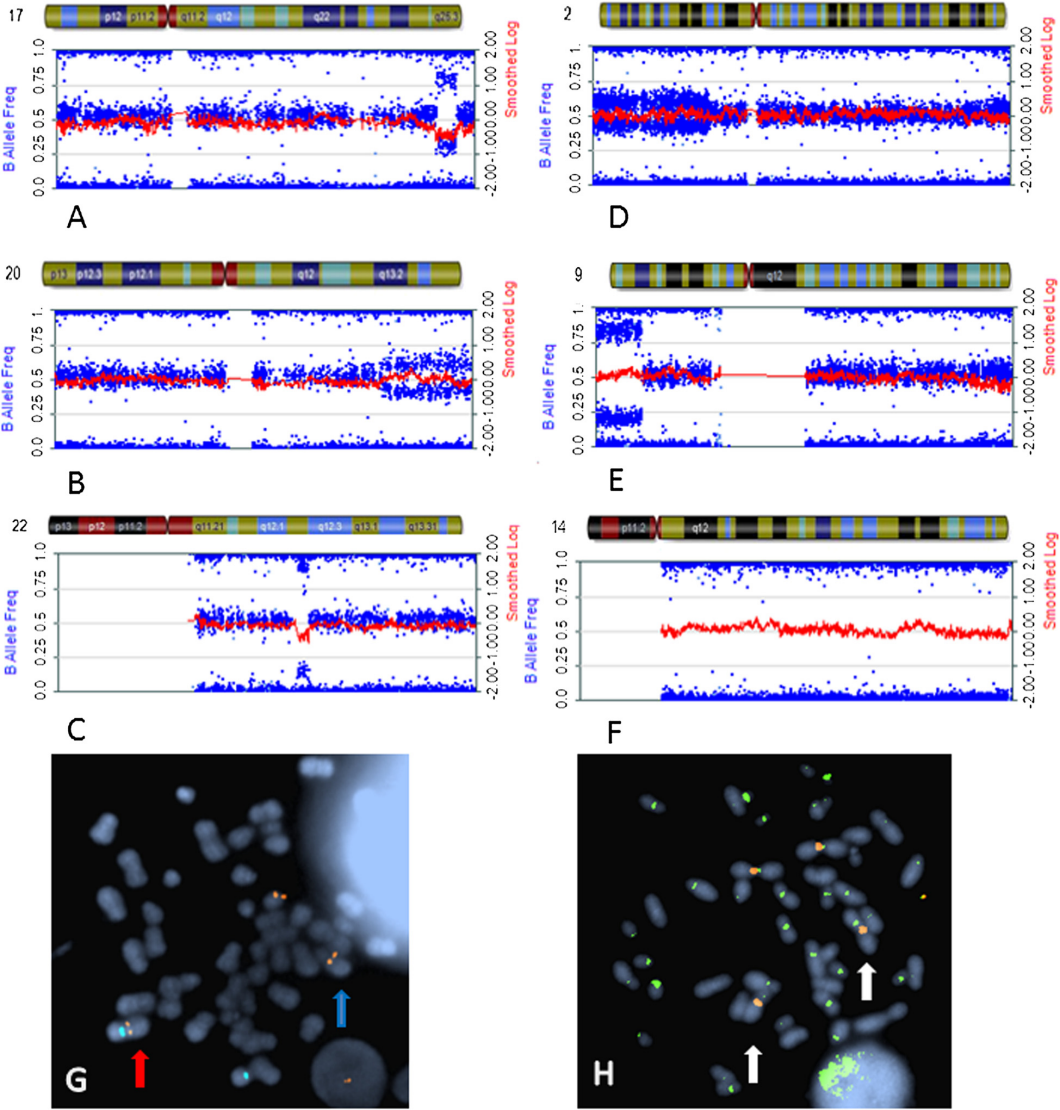
**Abnormalities detected by SNPa not observed by karyotyping.** SNPa profiles showing changes in B allele frequency (blue) and LogR ratio (red) consistent with **(A-C)** additional copy number changes not observed on the karyotype involving deletion on 17q and duplication on 20q from Case no. 2 and deletion on 22q from Case no. 5; **(D-F)** mosaic CN-LOH involving terminal 2p (Case no 9) and terminal 9p (Case no. 2) as well as CN-LOH of the entire 14q arm in 100% of DNA from Case no. 5; **(G-H)** metaphase FISH analysis of Case no. 2 showing the 20q subtelomere (orange signals) on the chromosome 18 (aqua signals) segment of the der(18)t(1;18) (red arrow) and also on the derivative 20 from the t(1;20) (blue arrow).

Additional copy number changes detected by SNPa but not visible by karyotyping included, in Case no. 2, deletion of a 3.9 Mb region on distal 17q within the q25.1-q25.3 interval that houses *SRSF2* known to harbour mutations with poor prognostic impact in PMF and myelodysplastic syndrome (MDS) [16]. In the same patient, duplication of a 13.8 Mb segment on distal 20q extended from q13.13 to qter (Additional file 4), including the mitotic kinase gene, Aurora Kinase A (*AURKA*) (Figure 3A,B). A 1.6 Mb interstitial deletion involving the 22q12.1-q12.2 region was detected by SNPa in Case no. 5 (Figure 3C) and included four cancer related genes: *MN1*, *NF2*, *CHEK2* and *EWSR1*. In Case no. 6, copy number gain at 16q12.1→q12.2 spanned *TOX3* (refer below). Case no. 9 with numerical ploidy changes alone shown by karyotyping (Additional file 2) revealed large stretches of mosaic CN-LOH on terminal 2p, 9p and 17q (Additional file 4, Figure 3D). In two cases, SNPa analysis identified CN-LOH involving part of 14q (Case no. 2, bands 14q24.2 to q31.3) or entire 14q (Case no. 5, bands q11.2 to q32.33) (Additional file 4, Figure 3E,F).

### Non-polyploid cohort

Eight non-polyploid cases were studied further by SNPa (NP-SNPa) (Additional file 4). Four cases showed a normal karyotype including one case with sole loss of the Y. Three cases showed an abnormal karyotype with sole del(13q), sole inv(3q) and loss of chromosome 18 as well as a marker chromosome respectively. Cytogenetic studies in one case failed to yield metaphase cells.

The eight NP-SNPa cases showed a median of two aberrations per sample (range: 0–10). Twenty genomic changes were detected overall. One sample showed a complex karyotype with 10 changes alone attributed to this one sample. Three samples showed no abnormalities by SNPa (Case nos. 25, 40 and 41). CN-LOH of 9p was detected in two cases including for one case as a sole abnormality (Case no. 21), and complex abnormalities on 6p were detected in a further two cases (nos. 20 and 22) encompassing large genomic regions (Additional file 4). The remaining abnormalities were found in single cases. Case no. 31 with a normal karyotype showed a large block of CN-LOH on 11q accompanied by copy number loss flanking the immediate proximal 11q region. Case no. 39 had an unsuccessful karyotype and showed the del(20q) and a 480Kb gain on 1p31.1 involving only the *NEGR1* gene. Interestingly, Case no. 22 with loss of chromosome18/+marker as the only karyotypic abnormality in two serial analyses showed significant additional changes on SNPa including duplication of 5p with concurrent loss of 5q, complex changes on 6p (*DEK*) and deletion of small regions within 6q24.1 (*NMBR*), 11q14.1 (*DGL2*) and 12q15 (*MDM2*), deletion of 17p (*TP53*) and multiple gains on 21q (*ERG, RUNX1)*. In addition, chromosome 18q showed CN-LOH involving the q11.2q12.1 region and concurrent deletion of 18q12→qter, with the *ASXL3* gene located at the 18q12 breakpoint junction. This latter finding most likely reflects composition of the marker chromosome detected on the karyotype. The disease progressed within the study period and the patient subsequently died.

Recurring changes observed in two or more samples across the eight patients analysed in the polyploid cases incorporating both SNPa and karyotyping data involved gains on 1q (six cases) as well as loss on 22q (two cases) and rearrangements of distal 6p (three cases) (Table 1). CN-LOH involving 6p (two cases), 14q (two cases), the commonly described CN-LOH9p (two cases) and amplification of 9p (one case) involving the *JAK2* locus in MPN were also detected using one or both of these methods. The non polyploid group showed recurring changes on 6p (two cases), 9p (two cases) and one case with 22q CN-LOH.

**Table 1:** Common abnormalities in the polyploid group on combining SNPa and karyotyping data

**Chromosome**	**Abnormality**	**Cytoband interval**	**No. of cases**
1	Gain	q21.1-q44	4
1	Gain	q21.1-q32.1	2
9	Gain/LOH	p22.3-p24.3	3
6p aberrations	Gain/loss/other	p12.3-p21.1	3
6p	LOH	p12.3-p22.2	2
22	Loss	q21.1-q21.2	2
14	LOH	q24.2-q31.3	1
14	LOH	q11.2-q32.33	1

### Fluorescent in situ hybridisation (FISH) confirms SNPa findings and highlights further complex changes

Selected additional abnormalities detected by SNPa were verified by FISH. In Case no. 2, FISH analysis on cultured cells showed mosaicism of 66% for loss on 17q and 44% for gain of 20q, and in Case no. 5, 90% for loss on 22q. The change in the SNPa B allele frequency (BAF) plots derived from granulocytic DNA corresponded to 65% (17q), 40% (20q) and 90% (22q) mosaicism (Figure 3, A-C), further emphasising the utility of SNPa in samples with heterogeneous cell populations. In Case no. 2, FISH analysis also showed that additional 20q material identified by karyotyping (Additional files 1, 2; Figure 1B) was located on the der(18) involved with the t(1;18) in an unusual three way translocation (Figure 3, G-H). In this rearrangement, the distal segment of 20q together with the translocated 1q was duplicated on the der(18p). The duplicated 20q region was 13.8 Mb in length (Additional file 2, Figure 3B). No deletion on 18p was evident by SNPa. The differing size of the FISH clones showing del(17q) and gain of 20q in Case no. 2 is indicative of clonal divergence.

### Centrosome studies

Centrosome studies on CD34 cells of five cases with MF showed no structural centrosome abnormalities (data not shown).

### Micronucleus studies

**Figure 4:**
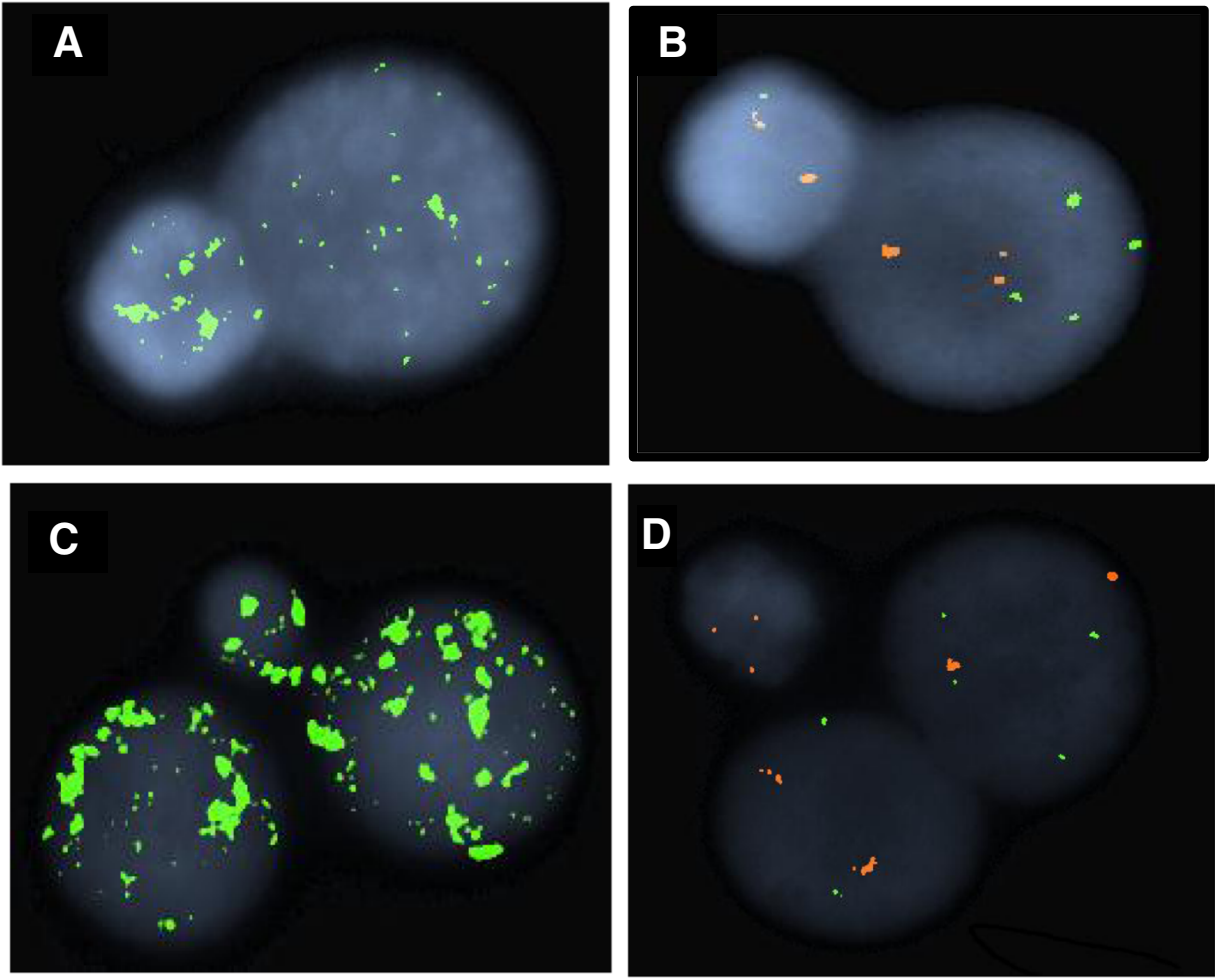
**Fluorescent in situ hybridisation analysis Cytochalasin B treated mononuclear cells from Case no. 1. A)** FISH image showing multiple chromosomes contained in the nuclear bulge using an all chromosome alpha satellite probe (green). **B)** two signals specific to the 1p arm (orange) and one signal specific to the 1q arm (green) present in the nuclear bulge while the larger nucleus showed four signal copies for 1q and two signal copies for 1p that correspond to the karyotype in Figure 1A (left image. **C)** FISH studies on binucleate daughter cells using an all chromosome alpha satellite FISH probe (green) showing expulsion of chromosome material from the binucleate cell to the micronucleus. **D)** FISH signal pattern for 1p and 1q arm probes showing unequal signal distribution of 1p and 1q in the two daughter nuclei and in the micronucleus.

Viable mononuclear cells were available from Case no. 1 for additional studies using the micronucleus assay. As illustrated in Figure 4, a proportion of mononuclear cells (60/1000 nuclei) showed chromosome expulsion by the formation of huge nuclear bulbs (Figure 4, A-B). Binucleate cells (15/500 binucleate nuclei) also showed expulsion of chromosomal material by the formation of micronuclei (Figure 4C). As shown in Figure 4D, unequal cell division was observed in binucleate daughter cells using FISH probes specific to 1p and 1q providing evidence for mitotic defects.

### JAK2V617F mutation studies, biological correlates and outcome

Five of nine polyploid cases (56%) showed the *JAK2* mutation (Additional file 4). Four were from the tetraploid group (Case nos. 1, 2, 4 and 6) and one from the mixed ploidy group (Case no. 9). Of the remaining 33 non-polyploid cases, 22 were *JAK2* mutation-positive (67%). Transformation to blast phase occurred in three of the nine polyploid cases and occurred within a median of 12 months from detection of myelofibrosis. Five patients showed disease symptoms associated with either a cytopaenia, bone marrow failure, progressive splenomegaly and/or karyotype evolution (Additional files 2 and 4). One patient (Case no. 9) remains in stable chronic phase. A Kaplan Meier estimate of overall survival between the polyploid group and the rest of the cohort of 42 patients showed a median survival of 4.7 years for the polyploid group and 9.7 years for the remainder of the group (data not shown). Two patients in the non polyploid group were lost to follow up. The log rank test showed no significant statistical difference between the two groups (*P* = 0.096). Tetraploidy was associated with a younger median age (54 years) relative to the whole cohort of 42 patients (68 years), and five of the nine patients with polyploidy underwent an allogeneic BM transplant. Two of the five patients survived at follow up (follow up range: 5 years and 12 months respectively). Two further cases received supportive care but died. In Case no. 3 with the der(6)t(1;6) the patient was in stable chronic phase for 16 years prior to the study but has since become transfusion dependent. Only one patient (Case no. 25) from the NP-SNPa group transformed to post-MPN blast phase while a further two cases (nos. 20 and 22) showed advanced disease with additional complex genetics changes by SNPa. All three patients subsequently died. The remaining five patients from the NP-SNPa group are alive including one who received a BM transplant and remains alive at 4 years post-transplant.

## Discussion

There have been few reports describing polyploid karyotypes in MPN. An 8n or near 8n karyotype was reported in one case each of MF and ET [17,18] and, more recently, a single case of near tetraploidy was recorded by Djordjevic et al. [11] in PMF. The increased detection of polyploidy in our cohort may be attributed to several factors, including: (i) the relatively low disease incidence and only recent inclusion of cytogenetic data in disease classification systems; (ii) the availability of sufficient numbers of metaphases for an extended analysis in order to define the presence of the polyploid clones and detailed follow-up cytogenetic studies that were not always achievable in reported cytogenetic studies done on fibrotic BM; iii) the possibility of a decreased sensitivity in some samples to the activity of spindle poisons leading to failure of mitotic arrest and the generation of polyploid metaphases, and iv) unique as yet unknown biological factors in the subject population.

Micronucleus analysis of one of our polyploid cases provided some insight into the pathophysiology of the polyploidy, showing elimination of chromosome material by the formation of micronuclei and large nuclear extrusions from mononucleate cells, a known mechanism of polyploid rescue [19].

Overall, our cytogenetic studies have shown a striking high frequency of gain of 1q material, evident in six of nine polyploid cases. Whereas gain of 1q has been found commonly in PMF as unbalanced 1q arm translocations or interstitial duplication, the association with polyploidy has not previously been reported in MPN [20-23]. Due to the large size of the genomic imbalances, identification of candidate genes on 1q in MPN has been difficult to ascertain and gene associations remain to be clarified. Recent reports in the literature have highlighted the role of the *MDM4* gene in MPN contained in the commonly duplicated 1q32 region [24,25]. The MDM4 protein binds directly with TP53 and acts as a negative regulator of *TP53*. Over expression of *MDM4* is a potent inhibitor of *TP53* leading to the accumulation of DNA damage and genomic instability. *PDE4DIP, PRCC* and *ABL2* on 1q close to the heterochromatin are associated with cell division, play a role in centrosome anchorage, mitotic checkpoint or microtubule binding and may be implicated in the development of polyploidy. Notably, the presence of chromosome aneuploidy or large chromosome imbalances are also known to give rise to neoplasia, genomic instability and polyploidy [15].

High hyperdiploidy (>67 chromosomes) in childhood acute lymphoblastic leukaemia (ALL) has been described in association with gain of 1q in 10-15% of cases and has been widely studied in haematological malignancies in relation to ploidy level and cytogenetic abnormalities. Gain of the 1q22-32.3 region was shown to be implicated and contains the *B4GALT3, DAP3, RGS16, MEM183A, and UCK2* genes [26,27]. Microdeletion studies by Paulsen et al. [28] using genome wide array analysis showed involvement of 7p12.2 (*IKZF1)*, 9p21.3 *(CDKN2A),* 9p13.2 *(PAX5)*, 12p13.2 (*ETV6)*, 13q14 *(RB1)*, 19p13.3 *(TCF3)* in association with hyperdiploid ALL. These regions bear some similarity to commonly affected regions reported in MPN [29]. In addition, gene mutations of *FLT3* (10-25%), *KRAS/NRAS* (15-30%) *and PTPN11 *(10-15%) have also been detected in hyperdiploid ALL [30]. In a different report, Paulson et al. [31] studied global epigenetic changes and found hyper methylation of the CpG regions of the *CADM1, ESR1, FHT, RARB* and *WNT5A* genes in more than 50% of hyperdiploid cases, showing a high propensity for epigenetic phenomenon in this ploidy group. The role of epigenetics in MF has been increasingly shown and the list of associated genes continues to grow although all the above genes have not been described in PMF [6,29].

Multiple myeloma is another haematological disease showing strong association with chromosome hyperdiploidy and frequent gain of 1q/dup1q as described in 59% of cases in the study by Marzin et al. [32]. Whole arm translocations were frequently associated with jumping translocations involving the whole 1q arm as the donor chromosome and involved the pericentromeric regions in 46% and the telomeric regions in 40% of recipient chromosomes. The proposed mechanism for the latter observation was based on the decondensation of the pericentromeric heterochromatin that favoured translocation to other sites of homologous repetitive sequences in the genome. Duplication of 1q occurred preferentially in the 1q21q22 and 1q31q44 regions in their study. This bears significance in regard to the mechanism related to the genetic instability and complex changes unmasked in Case no. 2 of our study that appear to involve translocation of the 20q subtelomere with the 1q heterochromatic region.

Of additional interest is the report by Silva et al. [33] on four infants with Down syndrome with gain of 1q that developed acute megakaryocytic leukaemia and provides some indication of a possible association between genes on chromosome 1q and megakaryocyte growth and development.

Abnormalities on 6p were a further frequent change, found in five of the 16 cases analysed using SNPa and involving gain, loss, translocation, CN-LOH or a combination (Additional file 4). Whereas the der(6)t(1;6) is a possible primary cytogenetic change in MF, the add(6p) and t(6;9) observed in our polyploid cases have been associated with a more progressive disease stage [34,35]. In support of these associations, two of our cases from the non-polyploid group with complex changes on 6p showed progressive MF. Three cases (two polyploid, one non-polyploid) showed SNPa/chromosome rearrangement possibly involving the *DEK* oncogene locus, known to be implicated in acute myeloid leukaemia and originally characterised as a result of *DEK/NUP214* fusion associated with the t(6;9) in AML. *DEK* has also been implicated in DNA damage repair and signalling [36].

Studies in the literature using SNPa show a marked variability and range in the types of genetic abnormalities detected in MPN. Despite this variation, a pattern of common changes is emerging as more studies are undertaken. Stegelman et al. [37] found no differences in the pattern of genetic abnormalities that could distinguish the classical MPN subgroups in 151 patients studied by SNPa. In that series, 45 patients were described with PMF and 14 patients with sMF. Recurrent copy number changes in PMF involved +1q, +8 and del(20q) in addition to CN-LOH on 9p, whereas common changes affecting sMF cases included +9, del(17q11.2), del(20q) and CN-LOH 9p. The remaining abnormalities detected were restricted to single cases [37]. Kawamata et al. [38] studied 16 patients with PMF and reported the del(13q), CN-LOH on 1p and CN-LOH on 9p as recurrent changes with the remaining abnormalities in single cases alone.

Of our 16 MF cases analysed using SNPa, CN-LOH was detected at one or more chromosomal regions in roughly equal proportions for both polyploid (3/8 cases) and non-polyploid (4/8 cases) cohorts. Although we found no particular association of specific CN-LOH regions with either group, some recurring observations are of note. These include CN-LOH of 9p in three cases, all overlapping the *JAK2* locus and all positive for the *JAK2V617F* mutation, an observation consistent with published findings [37,38]. CN-LOH also involved distinct regions of 6p in two cases, a finding of interest given the high frequency of 6p aberrations observed in our MF karyotypes overall, and by SNPa. In single cases, CN-LOH also affected other chromosomal regions, including 11q, 14q, 18q and 22q, all of which have been previously reported in isolated MF patients from different cohorts by others [25,37-40]. An interesting observation in our cases was the presence of genes involved in DNA damage repair and cell cycle regulation represented in most regions of CN-LOH, i.e. *MSH6, MSH2* (2p), *FANCE* (6p), *FANCG* (9p), *CHECK1* (11q), *RAD51L1* (14q) and *CHECK2* (22q). This may reflect an underlying defect in the DNA damage repair pathway and may explain the widespread nature of the genomic defects observed in MF.

Small regions of copy number aberration (loss, gain, structural interruption) identified using SNPa bring focus to individual genes as candidate oncogenes or tumour suppressor genes possibly underlying the biology of MF. Of our 16 MF cases analysed using SNPa, two showed deletions involving 11q14 and the *DLG2* gene, which encodes a scaffolding protein involved in cell signalling, and this was the sole gene affected in one case. *NEGR1* (gain-1p31.1), *NMBR* (loss*-*6q24.1), *TOX3* (gain-16q22) and *ASXL3* (interruption-18q21.1) were sole genes contained in regions of copy number change observed in single cases. *NEGR1* is implicated in cell adhesion, *NMBR* is a strong mitogen and growth factor implicated in solid cancers, the *TOX3* protein is involved with chromatin structure and plays a role in the unwinding and folding of DNA, and *ASXL3* belongs to the Additional Sex Combs-like family of three chromatin modifier genes of which *ASXL1* is commonly described in PMF [29].

Few of these genes have been completely characterised, and their role, if any, in MF or other haematological neoplasms remains to be determined. Nonetheless, as descriptions of clinical phenotypes associated with MF become increasingly refined, recurring alterations may eventually be associated with specific disease subsets. Towards this end, more recent studies have focused on the clinical stage associated with genetic abnormalities observed by SNPa [24,41]. In a series of 408 samples, Klampfl et al. [24] reported that changes involving 1q and 9p were strongly associated with sMF or progression to accelerated phase (AP) whereas, changes involving 1q, 3q, 5q, 6p, 7p, 7q, 19q, and 22q were associated with post-MPN AML when compared to chronic phase MPN. No associations were found by Klampfl and co-workers that distinguished sMF/AP from post MPN AML. In our series of nine polyploid MF cases, the common abnormalities detected on combining SNP and karyotype data included gains affecting 1q, 6p and 9p, and loss/LOH affecting 6p, 9p, 22q and 14q, findings that concur with the above earlier reports of non-polyploid patient series. These patterns of genetic changes are also similar to the SNPa profiles reported by Gondek et al. [39] that showed an overlap between MPN, MDS and MDS related AML [39,42].

In our series, disease evolution in the polyploid group occurred after a much shorter disease duration. This was reflected in patients both with and without structural karyotype abnormalities, with the exception of one case harbouring a der (6) t (1;6) who showed a disease duration of 16 years and who does not appear to follow the progressive disease course typical of patients with additional changes to 6p [41]. The monosomal karyotype (case no. 6) and the inversion 3 (case no. 25) were associated with a dismal outcome as reported previously by other investigators [43,44]. Two polyploid cases showed no abnormalities by either karyotyping or SNPa, a finding possibly reflecting the limitations of genomic resolution in our analysis or alternative disease mechanisms.

## Conclusions

In this study we found that SNPa with a medium density array platform using DNA purified from PB samples was sensitive to detect known and additional submicroscopic changes in MF, including CN-LOH and an assessment of the degree of clonal mosaicism. However, minor subclones, balanced rearrangements and polyploidy were not detected by SNPa. Overall, our findings suggest that polyploid subclones are more frequent in the PB of patients with MF than previously known and demonstrate that gain of 1q appears to be a common association.

## Methods

### Patients

Written consent was obtained from all patients according to the research protocol approved by the Northern Sydney Human Research Ethics Committee in accordance with the Declaration of Helsinki. Patient disease was classified according to current WHO recommendations [45]. Ten ml of PB was collected in heparin and EDTA on 42 consecutive patients diagnosed with MF (Additional file 1). In some cases, BM was additionally, or alternatively, collected for the purpose of routine patient care. Both sample types were collected at different time points through the disease course (Additional file 2).

### Chromosome analysis

Cultures were established from the buffy coat obtained from 10 ml of heparinised PB or from freshly aspirated BM. Cells were cultured for two days without mitogenic stimulation, metaphase cells harvested and slides prepared using standardised protocols [46]. Two independent harvests were carried out on each PB sample using colcemid (Life Technologies, USA) or vinblastine-colchicine (United Biosciences, QLD, Australia) at a final concentration of 2 μg/mL for 1 hour or 0.3 μg/mL overnight, respectively. BM cultures were harvested using only vinblastine-colchicine. Analysis of at least 20 GTL-banded metaphase cells was attempted on each sample. Cytogenetic studies were performed at diagnosis or at follow up on BM or PB samples submitted to the laboratory for routine clinical management. The ISCN 2013 cytogenetic nomenclature was used to describe the karyotype [47].

### CytoSNP12 array

Granulocytes from 10 ml of PB collected in EDTA were separated by Ficoll density gradient centrifugation. DNA was extracted from purified cells of eight MF patients with a polyploid subclone using the Qiagen Puregene kit (Valencia, CA). Case no. 4 was not further investigated by SNPa due to insufficient sample. Eight age- and sex-matched MF cases without polyploidy, selected from our total cohort of 42 cases, were also studied using SNPa for comparison. DNA was processed for the HumanCytoSNP-12 BeadChip assay by the Australian Genome Research Foundation according to the manufacturer’s instructions (Illumina, San Diego, CA). Data analysis was undertaken using GenomeStudio software version 2011.1 from Illumina. Copy number changes and CN-LOH were determined by analysis of the LogR ratio and the BAF plots generated by GenomeStudio. The median probe spacing on the array was 6Kb, and 20 consecutive markers were required for a copy number change to be called for a deletion or duplication. CN-LOH was called when a normal LogR ratio of 0 was obtained and a BAF score of 0 and 1 with no heterozygous calls at a BAF of 0.5. CN-LOH <5 Mb was excluded unless it contained known regions of significance to the sample population under study. Mosaicism was estimated by visual inspection of the BAF and compared to simulated plots as described by Nancarrow using the SIDCON method [48]. The genome browser used for analysis was set to build GRCh 37/Hg19 (http://genome.ucsc.edu). Common copy number variants were excluded if listed in the Database of Genomic Variants (http://dgv.tcag.ca) or if present when compared to the PB DNA of 12 healthy controls sourced from the HapMap sample data set provided by Illumina. Germline changes cannot be totally excluded as no matched germline patient DNA was available for study by SNPa. Gene prioritisation was undertaken with the aid of the Sanger Cancer Genome Census (http://www.sanger.ac.uk/cosmic). Sole genes in regions of change or genes described in published series relevant to PMF were also included.

### Fluorescent in situ hybridisation (FISH)

Directly labelled DNA probes specific to the chromosome 1q pericentromeric region and to alpha satellite regions of chromosomes 9 and 18 (Kreatech, Amsterdam, Netherlands) were used to confirm breakpoints on the derivatives 1q. The ON *EWSR1* (22q12) Break probe (Kreatech Diagnostics, Amsterdam, Netherlands), TelVysion 20q SpectrumOrange (Abbott Molecular, Des Plaines, IL), and BAC clones RP11-318A15 (17q25.1) and RP11-398 J5 (17q25.3) (The Centre for Applied Genomics, Toronto, Canada), were used to confirm SNPa findings. Probes were applied to fixed cells prepared for chromosome analysis from cultured PB buffy coat cells (refer above). The FISH procedure was carried out by standardised protocols as specified by the manufacturer with minor modifications. Briefly, slides were pre-treated in 10% (w/v) pepsin/0.1 N HCl for 13 minutes and probes hybridised overnight at 37°C. Post hybridisation procedures were carried out by washing slides in 0.1 XSSC, pH7.2/0.3% NP40 (v/v) at 72°C for 2 minutes and then in 2XSSC, pH7.2/0.1% NP40 at room temperature for 10 seconds. Slides were counterstained using DAPI (125 ng/mL, Abbott Molecular, Des Plaines, IL), and viewed under an Olympus BX61 fluorescence microscope equipped with excitation and emission filters appropriate to the probe fluorophores used.

### Centrosome studies

The technique for centrosome analysis was based on a modification of the method of Gisselsson et al. [14]. Cytospins were prepared from CD34+ cells enriched from PB buffy coat of five samples using microbead technology (Miltenyi Biotec, Bergisch Gladbach, Germany), fixed in 100% methanol for 20 minutes and then air dried. Slides were rehydrated in 1× phosphate buffered saline (PBS) for 10 minutes at room temperature, drained, then incubated in 200 μL of 1% (w/v) bovine serum albumin (BSA)/PBS for 15 minutes at 37°C in a humid chamber. After draining, 200 μL of 1% BSA/0.1% Triton X-100 (v/v)/PBS was applied for 15 seconds, the slide quickly drained, and 200 μL of the primary antibody against gamma tubulin, a centrosome specific protein (Santa Cruz Biotechnology, Europe), applied for 30–60 minutes at room temperature after diluting 1:40 (v/v) in 1% BSA/PBS. The slide was washed thrice in PBS for 5 seconds each. The goat anti-mouse IgG-FITC conjugate secondary antibody (sc-2080,Santa Cruz Biotechnology, Dallas, Texas, USA) was diluted 1:40 in 1% BSA/PBS, 200 μL applied and the slide incubated for 30–60 minutes at room temperature in the dark. Slides were then washed in PBS and air dried in the dark. Ten μL of DAPI (0.125 ng/μL) was applied as a counterstain and slides viewed immediately using the Olympus BX61 fluorescence microscope equipped with FITC emission filters for visualisation. Nuclei (n = 500) were scored for abnormalities in centrosome number and size.

### Micronucleus studies

Mononuclear cells at a density of 0.5 ×10^6^ cells/mL were cultured after Ficoll gradient separation from PB and incubated at 37°C. After 44 hours, cytochalasin B (Sigma Aldrich) was added to a final concentration of 6 μg/mL for a further 24 hours. The tubes were centrifuged to pellet the sample and the supernatant removed. Cells were washed in 5 mL of PBS and centrifuged. The supernatant was removed to 0.5 mL and the cell pellet carefully resuspended in 100% methanol. Slides were prepared from the cell pellet and stained in 10% (v/v) Leishman stain (POCD Healthcare, Sydney, Australia) for 9 minutes. Slides were mounted on coverslips and viewed by light microscopy. Nuclei (n = 1000) were scored for defects in mononucleate and binucleate cells using the criteria outlined by Fenech et al. [49].

### JAK2V617F mutation studies

*JAK2V617F* DNA mutation studies were carried out using a Taqman qualitative assay that required 100 ng patient DNA extracted from granulocytes and primers specific for the mutation. Methods were according to Kroger et al. [50].

### Statistical analysis

Survival curve analysis was undertaken using the Kaplan Meier method. The log rank test was used to measure the significance of survival curves obtained for the polyploid group compared to the whole cohort.

## Abbreviations

ALL: Acute lymphoblastic leukaemia; AP: Accelerated phase; BAF: B allele frequency; BSA: Bovine serum albumin; CN-LOH: Copy neutral loss of heterozygosity; ET: Essential thrombocythaemia; FISH: Fluorescent in situ hybridisation; MF: Myelofibrosis; MDS: Myelodysplastic syndrome; MPN: Myeloproliferative neoplasm; NP-SNPa: Nonpolyploid single nucleotide polymorphism array; PBS: Phosphate buffered saline; PET-MF: Myelofibrosis post essential thrombocythaemia; PPV-MF: Myelofibrosis post polycythaemia vera; PV: Polycythaemia vera; sMF: Secondary myelofibrosis; SNPa: Single nucleotide polymorphism array.

## Competing interests

The authors declare that they have no competing interests.

## Authors’ contributions

NS designed the study, performed experiments and wrote the manuscript. CM analysed data and wrote the manuscript. MK performed the *JAK2V617F* mutation studies. KW performed the survival curve analysis. WS and CW reviewed the data and wrote the manuscript. All authors read and approved the final version of the manuscript.

## Supplementary Material

Additional file 1 **Patient characteristics and karyotypes of 42 consecutive cases.** File contains case ID, disease classification, JAK2 mutation status, sex, age and karyotype of entire cohort. Click here for file

Additional file 2 **Serial karyotype analysis of polyploid group.** File contains dated serial karyotypes of the nine polyploid cases. Click here for file

Additional file 3 **Base pair co ordinates and chromosome location of copy number changes.** File lists all genetic co ordinates of changes detected by SNP array. Click here for file

Additional file 44 **SNP array findings in 16 patients with myelofibrosis.** Tabular data combining patient disease characteristics, karyotype and corresponding changes detected by SNP array in addition to disease stage, subsequent therapy and survival outcome. Table also lists associated genes of interest. Click here for file
